# COMMD3 Expression Affects Angiogenesis through the HIF1*α*/VEGF/NF-*κ*B Signaling Pathway in Hepatocellular Carcinoma *In Vitro* and *In Vivo*

**DOI:** 10.1155/2022/1655502

**Published:** 2022-09-02

**Authors:** Tingting Zhu, Xiaolin Peng, Ziwei Cheng, Xiuru Gong, Dongwei Xing, Wei Cheng, Minguang Zhang

**Affiliations:** Shanghai Municipal Hospital of Traditional Chinese Medicine, Shanghai University of Traditional Chinese Medicine, Shanghai, China 200071

## Abstract

**Background:**

High expression of copper metabolizing MURR1 domain (COMMD3) is significantly correlated with poor prognosis in hepatocellular carcinoma (HCC) patients. Here, we explored the mechanism by which COMMD3 affects HCC angiogenesis through the HIF1*α*/VEGF/NF-*κ*B signaling pathway.

**Methods:**

SK-Hep1 and Hep-3B cell lines were transfected by *COMMD3* overexpression and RNA interference lentivirus and verified using RT-qPCR and western blotting techniques. Using RNA sequencing, we analyzed differentially expressed genes in *COMMD3*-overexpressed and *COMMD3*-knockdown HCC cells. Altogether, colony formation assay, wound healing assay, transwell cell invasion assay, flow cytometry apoptosis experiments, HUVEC tube formation detection, phalloidin staining assay, western blotting, immunohistochemical staining, and a nude mouse xenograft model were used for experimental verification.

**Results:**

Lentivirus *COMMD3* overexpression and knockdown were successfully established in HCC cells. *COMMD3* overexpression significantly promoted the proliferation, angiogenesis, migration, and invasion capacities of HCC cells with no obvious effect on apoptosis versus controls while *COMMD3* knockdown showed the opposite trend. The expression and protein levels of COMMD3 as well as HIF1*α*, VEGF, and NF-*κ*B were increased in *COMMD3*-overexpressing HCC cells versus control cells, while they were reduced after *COMMD3* knockdown. In addition, RNA-seq indicated that *COMMD3* is an indispensable gene for HCC angiogenesis through HIF1*α* and NF-*κ*B signaling pathways.

**Conclusion:**

This study showed that low expression of COMMD3 can inhibit HCC angiogenesis by suppressing the HIF1*α*/VEGF/NF-*κ*B pathway. This implicates COMMD3 as a potential biomarker for improving the therapeutic outcome of HCC.

## 1. Introduction

According to epidemiological studies, hepatocellular carcinoma (HCC) is the seventh most prevalent malignancy and the second leading cause of cancer-related deaths around the world [1, 2]. As a solid tumor, HCC is highly vascularized requiring angiogenesis for invasion and metastasis [3]. Without angiogenesis, HCC does not grow more than 1–2 mm [4]. Tumor formation creates a hypoxic microenvironment that activates hypoxia-inducible factor 1*α* (HIF1*α*) and nuclear transcription factor-*κ*B [5, 6], ultimately triggering vascular endothelial growth factor A (VEGF-A) secretion and the subsequent angiogenesis [7]. In recent years, antiangiogenesis therapy for HCC has made significant progress in clinical practice. Drugs targeting angiogenesis, such as sorafenib, have been investigated for mechanism in clinical practice, but their adverse reactions seriously affect the quality of life of patients [8]. The rapid advances in gene technology have deepened our understanding of angiogenesis, with research focused on finding new gene targets and better treatment options for HCC therapy.

There are ten members of the copper metabolizing MURR1 domain (COMMD) family of proteins, i.e., COMMD1–10 [9]. COMM domain is the main feature of COMMD protein that contains a conserved sequence of 70-85 amino acids. This domain is located at the carboxyl-terminal of the last protein and provides a platform for protein-protein interaction [10, 11]. COMMD family members are involved in many physiological activities of cells such as copper (Cu) homeostasis, sodium (Na) homeostasis, hypoxia responses, and endosomal adaptation, as well as inflammatory activities [11, 12]. Specifically, current studies have found that COMMD3 can promote the proliferation of HCC and be used as an independent prognosticator in HCC patients [13].

Angiogenesis is a complex process in tumor progression. Targeted angiogenesis is regulated by multiple signaling pathways, among which hypoxia is indispensable. Rius et al. [14] demonstrated that the nuclear factor of transcription kappa B (NF-*κ*B) is an essential mediator of HIF1*α* transcription. Under hypoxia, the NF-*κ*B pathway is activated and HIF1*α* expression is upregulated. It has been found that COMMD1, another member of the COMMD3 family, can inhibit downstream gene expression mediated by the NF-*κ*B pathway via blocking the HIF1*α* and HIF1*β* isodimer formation pathway, in turn inhibiting the malignant progression of tumor cells [15]. However, the relationship between COMMD3 and tumor angiogenesis has rarely been studied. Therefore, we used lentivirus transfection technology and transcriptome sequencing technology to evaluate the angiogenesis mechanisms of COMMD3 and HCC angiogenesis.

## 2. Methods

### 2.1. Cell Culture

Two HCC cell lines (SK-Hep1 and Hep 3B) and human umbilical vein endothelial cells (HUVECs) were acquired from the Chinese Academy of Sciences cell bank (Shanghai, China). The three cell lines were cultured as monolayers at 37°C in a humidified atmosphere with 5% CO_2_ pressure as previously described [16].

### 2.2. Cell Transfections

The slow virus vector was constructed by Genechem (Shanghai, China). SK-Hep1 and Hep-3B cells were seeded in 6-well plates to achieve 30~40% confluency on the next day. Then, following the manufacturer's instruction, sh*COMMD3*, ov*COMMD3*, or negative control (shCtrl) lentiviruses were transfected into the cells and cultured for 8–12 h with fresh complete media. The cells were monitored using a fluorescence microscope three days postinfection. Finally, stably transfected cell lines were screened with a puromycin-containing medium (2 g/mL). The short hairpin RNA (shRNA) sequences for targeting *COMMD3* included 5′-ACTCCAACGCCTTCACGCTTC-3′ and 5′-GGATGATCTAACACGGCCTCGTC-3′.

### 2.3. RNA-seq Analysis

Fully transfected *COMMD3* SK-HEP1 cells with overexpression and knockdown and wild-type SK-HEP1 cells were sent to Oebiotech (Shanghai, China) for total RNA extraction and total transcriptome sequencing. We used Gene Ontology (GO) enrichment analysis and gene set enrichment analysis (GSEA) to analyze the function and signaling pathway of the differentially expressed genes (DEGs). GSEA was specifically used to examine the relationships between stress responses in multiple cancer-related pathways, metabolic pathways, transcriptional processes, and biological processes.

### 2.4. Colony Formation Assay

After two weeks of culturing, 800 cells/well in 6-well plates were fixed using 4% paraformaldehyde. Then, the cell staining was performed using 0.2% crystal violet (Beyotime, Shanghai, China) for visualization, and individual colonies (>50 cells/colonies) were counted.

### 2.5. Wound Healing Assay

Briefly, when the cells in the 6-well plates reached 80–90% confluency, the cell monolayer was scratched with a 200 *μ*L plastic pipette tip, then washed. Subsequently, cells were cultured in a complete medium for 24 h. The healed wound area was measured at the beginning, and 24 h later, we used an inverted microscope to assess cell migratory abilities.

### 2.6. Transwell Invasion Assay

Transwell cell invasion was evaluated as described before [16]. Briefly, a 24-well transwell plate and an 8 *μ*m filter (Corning, NY, USA) were used. 50 *μ*L Matrigel was added to the upper cavity before cells, and 500 *μ*L containing 20% FBS medium was added to the lower lumen. Then, the invading cells were counted after 48 h.

### 2.7. HUVEC Tube Formation Detection

HUVEC tube formation was detected as described before [16]. Briefly, cells were incubated in 96-well plates with Matrigel at 37°C for 30 min. Subsequently, 100 *μ*L/well HUVEC cell suspension was added. The cell suspension contained 2.5 × 10^4^ HUVECs and the cell culture supernatant for each group. Then, cells were incubated for a further 4-6 h in a 37°C and the lumen formation was observed under a microscope. Finally, for fluorescent staining, calcein (Invitrogen, Carlsbad, CA, USA) was added to cells and incubated for another 30 min; then, cells were imaged and analyzed using a fluorescence microscope.

### 2.8. Phalloidin Staining Assay

A phalloidin staining kit (YEASEN, Shanghai, China) was used to stain the actin stress fibers following the manufacturer's instructions. Briefly, cells were grown on coverslips and fixed with 4% paraformaldehyde; then, 50-100 *μ*L cyclopeptide working solution and DAPI solution were added. Finally, the micrographs were photographed.

### 2.9. Apoptosis Assay

Cells were cultured at a density of 5 × 10^5^ per well in a 6-well plate and incubated overnight. On the next day, they were collected using EDTA-free trypsin and counted. Then, the apoptosis rate was evaluated using a PE Annexin V Apoptosis Detection Kit with a 7-AAD kit following the manufacturer's instructions (Tonbo Biosciences, San Diego, CA, USA). First, 5 *μ*L 7-AAD was added to each group. Then, 95 *μ*L 1 × 7-AAD binding solution was added before mixing, and finally, 5 *μ*L propidium iodide was added before incubating at 25°C in the dark for 15 min. Finally, flow cytometry (Cytomics FC 500; Beckman Coulter, Brea, CA, USA) was used for measuring the apoptosis rate.

### 2.10. Western Blotting (WB) Assay and qPCR Detection

WB and qPCR were performed referring to our previous articles [16]. Total RNA extraction was performed by the TRIzol™ reagent method. Per sample, 0.5 *μ*g total RNA was used as a template in an RT-qPCR kit (Takara, Shiga, Japan). Primer synthesis and purification were performed by Sangon Biotech (Shanghai, China) according to the following sequences: *COMMD3* forward (5′-ACTCCAACGCCTTCACGCTTC-3′), *COMMD3* reverse (5′-GGATGATCTAACACGGCCTCGTC-3′), GAPDH forward (5′-CAGGAGGCATTGCTGATGAT-3′), and GAPDH reverse (5′-GAAGGCTGGGGCTCATTT-3′). For WB, the 1 : 1,000 dilutions of primary antibodies were used, and then, the membrane was incubated overnight at 4°C. HIF1*α*, VEGF, NF-*κ*B, p65, *β*-Catenin, p-VEGFR2, VEGFR2, and actin antibodies were provided from CST (Danvers, MA, USA). COMMD3 antibody was provided from Abcam (Cambridge, UK).

### 2.11. Animal Studies

In total, 24 4-week-old male BALB/c nude mice were provided by Shanghai Slack Laboratory Animal (China) and divided into four groups (*n* = 6 per group). Each group received either normal SK-Hep1 cells, transfected SK-Hep1 cells with no load, stably infected Ov*CommD3* SK-Hep1 cells, and Sh*CommD3* SK-Hep1 cells subcutaneously injected into the right axilla (5 × 10^6^ cells treated with 100 *μ*L PBS). We evaluated tumor diameter 10 days after administration and every 3 days until the 27th day following the injection. Then, the tumor volume *V* (mm^3^) was calculated using the following formula: *V* = *L* × *W*^2^/2, where the *L* and *W* parameters represent the largest diameter (mm) and the smallest diameter (mm), respectively. On the 27^th^ day after implantation, the mice were sacrificed, and the tumor weight was measured. Then, tumors were subjected to immunohistochemical (IHC) staining and WB ex vivo as previously described [16]. This study was performed under the ethical approval of the Animal Ethics Committee of Shanghai Municipal Hospital of Traditional Chinese Medicine, Shanghai University of Traditional Chinese Medicine (Approval Number: SYXK, Shanghai, China 2020-0014).

### 2.12. Statistical Analyses

All experiments were performed in triplicate at least. Data are expressed as the mean ± SEM. For comparing the results, the means of multiple groups were compared by one-way analyses of variance (one-way ANOVA) in Prism v8.0 (GraphPad Software, San Diego, CA, USA). A *P* value < 0.05 was considered statistically significant.

## 3. Results

### 3.1. COMMD3 Overexpression and RNA Interference Lentivirus Stable Strains Were Successfully Constructed

**Figure 1:**
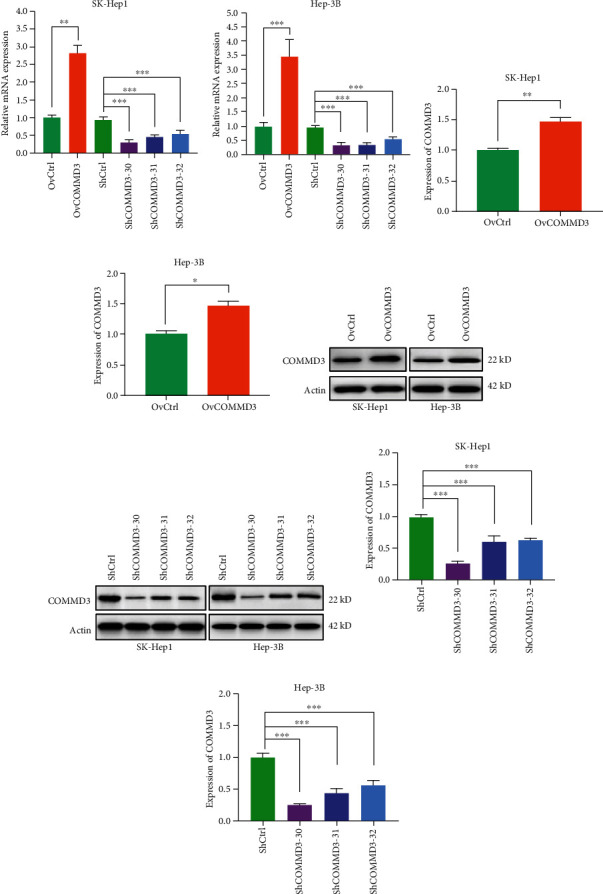
*COMMD3* expression and protein level after *COMMD3*-overexpression or *COMMD3*-knockdown in HCC cell lines. (a–b) Relative quantity of *COMMD3* mRNA level in SK-Hep1 and Hep-3B cell lines measured by RT-qPCR after *COMMD3* overexpression or knockdown. (c–d) COMMD3 protein levels increased after *COMMD3*-overexpressed transfection. (h–g) COMMD3 protein levels decreased after *COMMD3*-knockdown transfection. (e–f) Western blot of COMMD3 levels. Actin was used as the loading control. ^∗^*P* < 0.05, ^∗∗^*P* < 0.01, and ^∗∗∗^*P* < 0.001.

HCC cells were infected and subcultured. The stably transfected cell lines were screened by puromycin. The expression of *COMMD3*-mRNA in lentivirus-infected stable strains was measured by RT-qPCR. The outcomes indicated the significantly elevated expression levels of *COMMD3*-mRNA in *COMMD3* overexpressed cells compared to the negative control group. *COMMD3*-mRNA expression level after *COMMD3*-RNA interference was significantly lowered compared to the negative control group (Figures 1(a) and 1(b)).

We used WB to explore the protein levels of COMMD3 in stable strains infected with lentivirus. The results indicated that *COMMD3* overexpression increased the COMMD3 protein levels compared to the negative control group. However, the COMMD3 protein levels after *COMMD3*-RNA interference were significantly decreased compared to in the negative control group (Figures 1(c)–1(h)). Meanwhile, the *shCOMMD3*-30 sequence showed the highest knockdown efficiency among the three knockdown sequences of 30, 31, and 32. Therefore, we chose *shCOMMD3*-30 for the follow-up study (Figures 1(c)–1(h)).

These results indicate that *COMMD3* overexpression and RNA interference lentivirus stable strains were successfully constructed and used for the mechanism studies.

### 3.2. COMMD3 Modulates the DEGs among OvCOMMD3, Control, and ShCOMMD3 SK-HEP1 Cells

**Figure 2:**
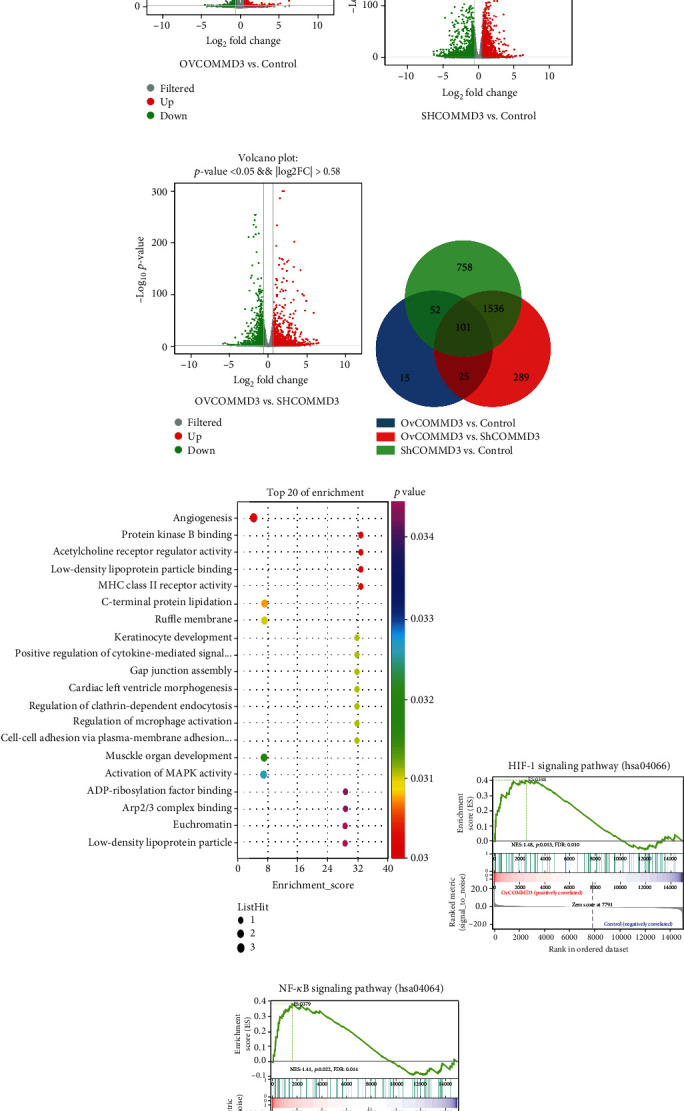
*COMMD3* modulates the DEGs among Ov*COMMD3*, control, and Sh*COMMD3* in SK-Hep1 cells. (a–c) The volcanic maps indicated the DEGs among the three comparative groups. The red color represents upregulated DEGs, the blue color represents downregulated DEGs, and the gray color represents the non-DEGs; log_2_FC ≥ 1.5 and *P* < 0.001. (d) Venn diagram of DEGs in control vs. Ov*COMMD3*, control vs. Sh*COMMD3*, and Sh*COMMD3* vs. Ov*COMMD3*; log_2_FC ≥ 1.5 and *P* ≤ 0.01. (e) Bubble diagram showed GO functional enrichment analysis. The *Q*-value is the calibration value for the *P* value. (f–g) GSEA for the HIF1*α* and NF-*κ*B pathway. A nominal *P* < 0.05 and false discovery rate (FDR) < 0.25 were set as statistically significant thresholds.

Ov*COMMD3*, control, and Sh*COMMD3* SK-HEP1 cells were detected by RNA-seq analysis. First, according to gene expression in each sample, 193 DEGs (68 upregulated DEGs and 125 downregulated DEGs) were found between Ov*COMMD3* and control SK-HEP1 cells (Figure 2(a)). Among the DEGs of Sh*COMMD3* and control SK-HEP1 cells, a total of 2,447 DEGs were affected (1,254 upregulated DEGs and 1,193 downregulated DEGs) (Figure 2(b)). In addition, there were a total of 1,951 DEGs between Ov*COMMD3* and Sh*COMMD3* (949 up- and 1,002 downregulated) (Figure 2(c)). The logarithmic fold-change (log_2_FC ≥ 1.5) and *q* − value ≤ 0.05 were used as the screening conditions. Also, a Venn diagram was used for showing 101 common DEGs among the three groups (Figure 2(d)). In addition, using the GO functional enrichment analyses, we showed that *COMMD3*-enriched genes were mainly involved in angiogenesis processes (*P* < 0.05; Figure 2(e)). GSEA displayed that differentially expressed Ov*COMMD3* and control genes were enriched in the HIF1*α* (Figure 2(f)) and NF-*κ*B signaling pathways (Figure 2(g)) (*P* < 0.05, FDR < 0.25).

### 3.3. COMMD3 Expression Affects the Formation of Clonal Plaques of Human HCC Cells

**Figure 3:**
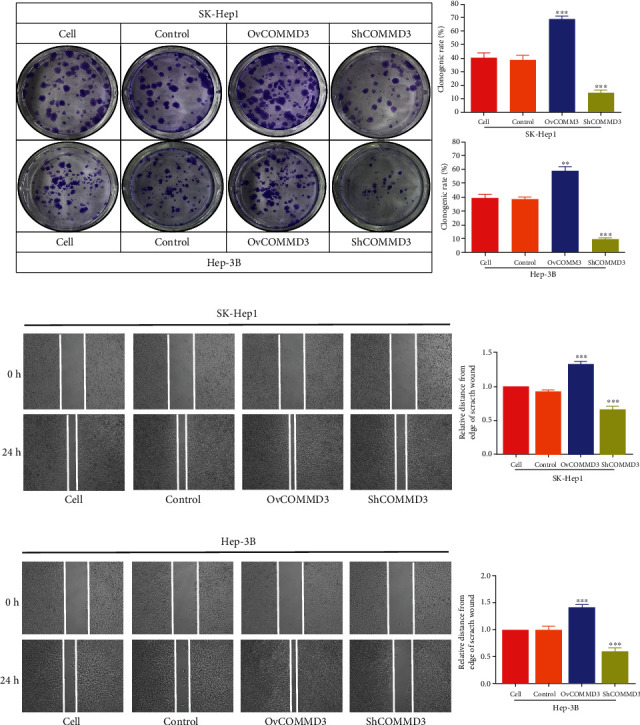
*COMMD3* regulates the proliferation and migration of HCC cell lines. (a) Clone formation assay showing the proliferation capacity of both HCC cell lines after *COMMD3* overexpression or knockdown. (b) Wound healing assessment displaying the migration capacity of SK-Hep1 and Hep-3B cell lines after *COMMD3* overexpression or knockdown. ^∗∗^*P* < 0.01; ^∗∗∗^*P* < 0.001.

The colony formation assay after *COMMD3* overexpression showed an enhanced clone formation capacity in HCC cells. This finding evidenced the stronger proliferation ability of these cells compared to the control ones (*P* < 0.001 for both cell lines). However, after *COMMD3* knockdown, the plate clonal plaque formation ability showed a significant reduction compared to the control cells (*P* < 0.001 for both cell lines) (Figure 3(a)). These results show that *COMMD3* overexpression enhances the proliferation capacity of HCC cell lines, whereas *COMMD3* knockdown reduces their proliferation potential.

### 3.4. COMMD3 Expression Affects the Migration of HCC Cells

In both HCC cell lines with *COMMD3*-RNA interference, the number of migrating cells to the opposite side was less than that of the control cells 24 h after the scratch. In other words, the scratch area of both HCC cell lines was larger than that of the control group (*P* < 0.001 for both cell lines). However, in the two HCC cell lines with *COMMD3* overexpression, the area of the scratch remained smaller compared to the control group (*P* < 0.001 for both cell lines) (Figures 3(b) and 3(c)).

### 3.5. COMMD3 Expression Affects the Invasion Capacity of HCC Cells

**Figure 4:**
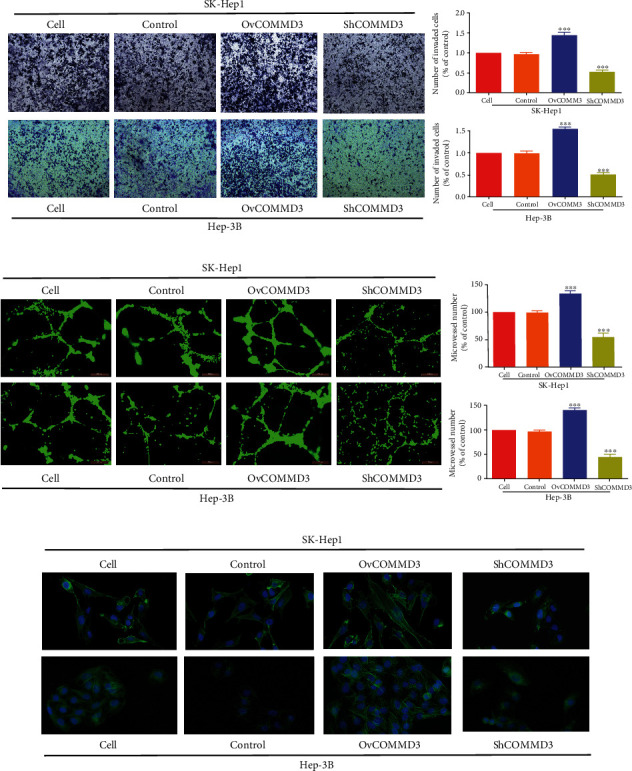
*COMMD3* regulates invasion and angiogenesis of HCC cell lines. (a) Transwell assay showing the invasion capacity of HCC cell lines after *COMMD3* overexpression or knockdown. (b) HUVEC tube formation detection of the angiogenesis capacity of HCC cell lines after *COMMD3* overexpression or knockdown. (c) Phalloidin staining assay of the F-scaffolding protein actin of HCC cell lines after *COMMD3* overexpression or knockdown. ^∗∗∗^*P* < 0.001.

Based on the transwell cell invasion experiment, the *COMMD3*-overexpressed HCC cells showed stronger invasive ability than control cells after 48 h (*P* < 0.001 for both cell lines). On the contrary, after *COMMD3*-knockdown, the invasion ability was significantly attenuated compared to the control cells (*P* < 0.001 for both cell lines) (Figure 4(a)).

### 3.6. COMMD3 Expression Affects the Angiogenesis of HCC Cells

According to Figure 4(b), the angiogenesis capacity of the *COMMD3*-overexpressing SK-Hep1 and Hep-3B cell lines was significantly higher than that of the control ones (*P* < 0.001 for both cell lines). However, RNA interference inhibited the angiogenesis capacity in both cell lines (*P* < 0.001 for both cell lines).

### 3.7. COMMD3 Expression Affects Actin Microfilament Morphology of HCC Cells

Phalloidin staining is used to mark the F-scaffolding protein actin in cells. The F-actin distribution is extensive when the angiogenesis ability of cells is weakened. In contrast, when angiogenesis is enhanced, F-actin distribution is dispersible. As shown in Figure 4(c), the cytoskeleton of the control groups formed a linear pattern assembly in parallel throughout (*P* < 0.001 for both cell lines). However, cells with *COMMD3* overexpression had densely distributed F-actin in the cytoskeleton, while *COMMD3*-RNA interference resulted in the dispersion of disordered or disappearing stress fibers, indicating that *COMMD3*-RNA interference induced F-actin depolymerization and weakened angiogenesis ability (*P* < 0.001 for both cell lines).

### 3.8. COMMD3 Expression Affects the Apoptosis Capacity of Human HCC Cell Lines

**Figure 5:**
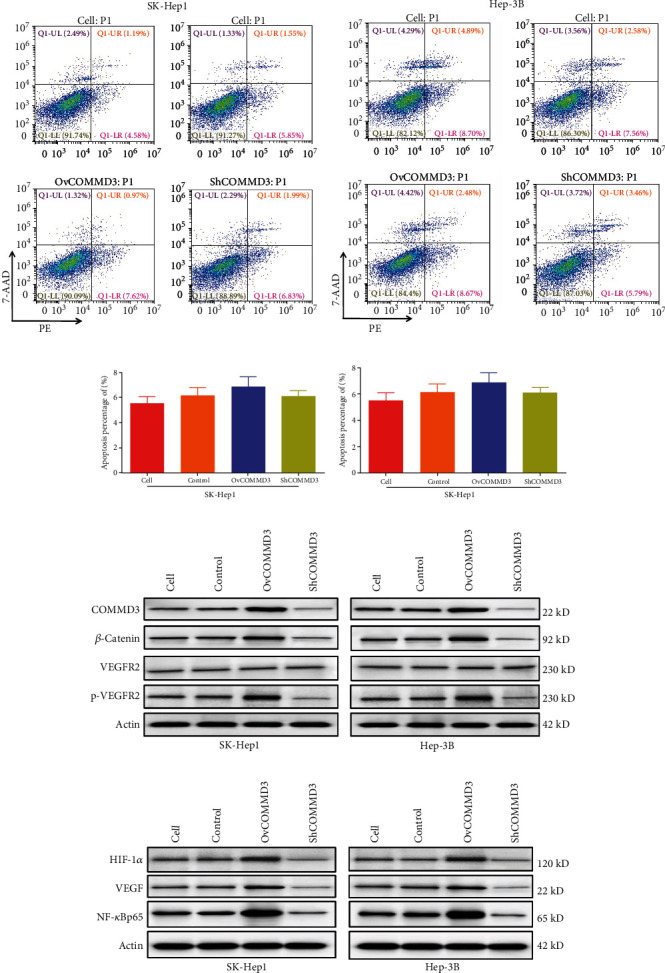
*COMMD3* regulated the protein expression levels of *β*-Catenin, *p*-VEGFR2, VEGFR2, HIF1*α*, VEGF, and NF-*κ*B, without affecting apoptosis in HCC cell lines. (a–d) *COMMD3* expression had no significant effect on apoptosis of HCC cell lines. (e–f) Western blot for *β*-Catenin, *p*-VEGFR2, VEGFR2, HIF1*α*, VEGF, and NF-*κ*B levels.

In the apoptosis experiment using flow cytometry (Figures 5(a)–5(d)), after *COMMD3* overexpression and *COMMD3* knockdown, the apoptotic behavior of HCC cell lines did not significantly change compared to the control cells (*P* > 0.05 for both cell lines). Therefore, *COMMD3* expression showed no statistically significant effect on the apoptotic behavior of HCC cell lines.

### 3.9. COMMD3 Expression on *β*-Catenin and p-VEGFR2/VEGFR2 Protein Levels in HCC Cells

To explore the key factors involved in COMMD3 promoting angiogenesis in SK-Hep1 and Hep-3B cell lines, we used WB to explore the protein levels of *β*-Catenin and *p*-VEGFR2/VEGFR2 in *COMMD3*-overexpressed and *COMMD3*-RNA interference cells. According to the results (Figure 5(e)), *COMMD3* overexpression increased *β*-Catenin and *p*-VEGFR2/VEGFR2 protein levels compared with the controls, while *COMMD3*-RNA interference suppressed these protein levels. These findings evidence that COMMD3 can enhance angiogenesis in HCC cell lines through activating *β*-Catenin and promoting the phosphorylation of VEGFR2.

### 3.10. COMMD3 Expression on the Levels of HIF-1*α*, VEGF, and NF-*κ*B in HCC Cell Lines

The signaling pathways involved in COMMD3 promoting migratory, invasiveness, and angiogenic properties of HCC cell lines were explored along with the GSEA results. For this purpose, we used WB to evaluate the protein levels of HIF1*α*, VEGF, and NF-*κ*B in the HCC cell lines of *COMMD3* overexpression and *COMMD3*-RNA interference. The *COMMD3*-overexpressed cells produced higher protein levels of HIF1*α*, VEGF, and NF-*κ*B compared with the control cells. However, the expression of these proteins was inhibited in the *COMMD3*-RNA interference group compared to the control cells (Figure 5(f)).

The present findings indicate that *COMMD3* can promote HIF1*α*, VEGF, and NF-*κ*B protein levels in HCC cell lines. Considering our results, the expression of *HIF1α*, *VEGF*, and *NF-κB* is upregulated in HCC, which promotes cancer cell migration and induces angiogenesis. Therefore, we postulate that *COMMD3* can promote the migratory, invasiveness, and angiogenic properties of HCC cell lines regulating the HIF1*α*/VEGF/NF-*κ*B signaling pathway.

### 3.11. COMMD3 Regulates Tumor Growth in a Xenograft Mouse Model

**Figure 6:**
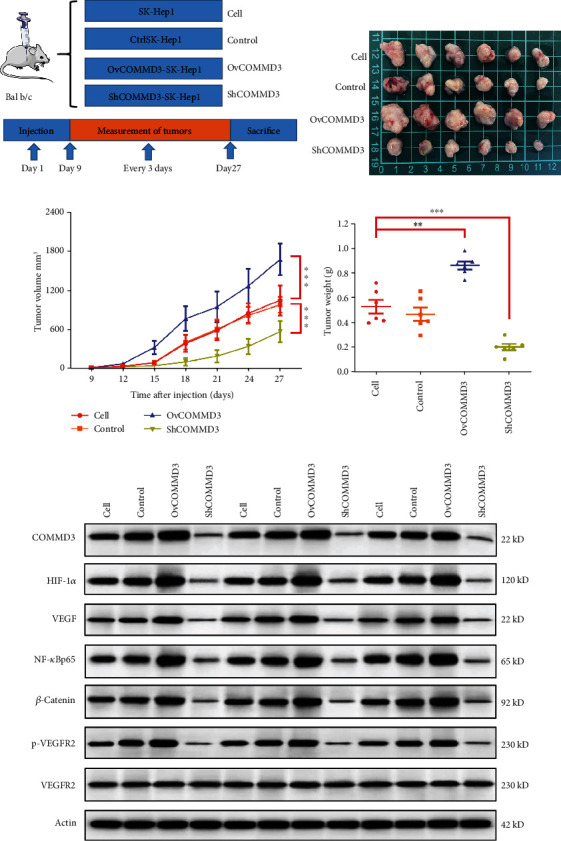
Effects of *COMMD3* on subcutaneous tumor growth and angiogenesis in nude mice. (a) Schematic diagram of animal experimental groups and time points. (b–d) Effects of COMMD3 on tumor progression. (e–f) Western blot for *β*-Catenin, *p*-VEGFR2, VEGFR2, HIF1*α*, VEGF, and NF-*κ*B levels. Actin was applied as the loading control. ^∗∗^*P* < 0.01; ^∗∗∗^*P* < 0.001.

To verify the role of *COMMD3* in the angiogenesis of HCC, we conducted experiments on transplanted tumors in nude mice. For this purpose, SK-Hep1 (as control), *COMMD3*-overexpressing cells, and *COMMD3*-knockdown cells were administrated into the nude mice subcutaneously (Figure 6(a)) to form a subcutaneous xenograft tumor model. Figures 6(b)–6(d) represent the nude mouse models subcutaneously transplanted with SK-Hep1 control cells, exhibiting a considerable tumor growth rate. The tumor growth rate and tumor weight in mice administered with the *COMMD3*-overexpressing cells showed a significant increase compared to the control group (*P* < 0.001). Conversely, the *COMMD3*-knockdown group represented with decreased tumor growth rate and tumor weight (*P* < 0.001).

### 3.12. COMMD3 Modulates Angiogenesis in Subcutaneous HCC Xenograft Mice

**Figure 7:**
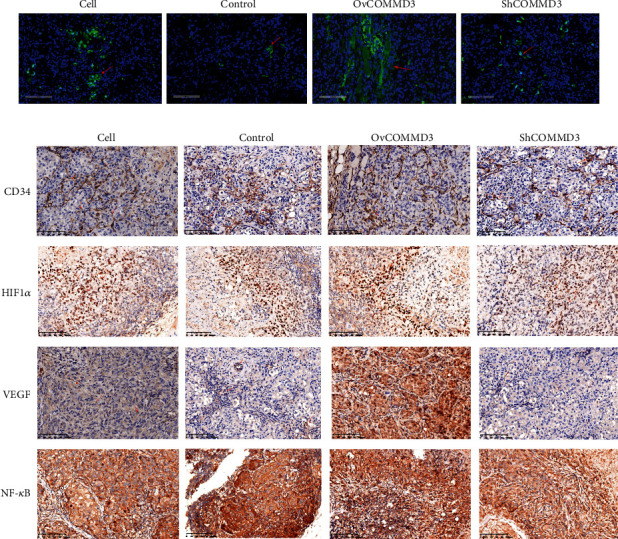
*COMMD3* modulates the F-scaffolding protein actin and the levels of CD34, HIF1*α*, VEGF, and NF-*κ*B in nude mice. (a) Phalloidin staining assay of the F-scaffolding protein actin in nude mice. (b) Immunohistochemical staining for CD34, HIF1*α*, VEGF, and NF-*κ*B levels.

For detailing the function mechanism of *COMMD3* in regulating the growth of subcutaneous HCC xenograft tumors in nude mice, tumors were studied histologically. For this purpose, the tumors were collected after the nude mice were sacrificed, and the phalloidin staining was used to explore the F-actin of the tumor cells. As shown in Figure 7(a), F-actin was densely distributed in the *COMMD3*-overexpressing cytoskeleton, while F-actin was scattered in the *COMMD3*-RNA interference cytoskeleton. The results suggested that *COMMD3*-overexpression promoted F-actin aggregation and enhances angiogenesis, while *COMMD3*-RNA interference induced F-actin depolymerization and weakened angiogenesis.

The tumor was also tested by IHC staining to explore the protein expression levels of angiogenesis-related *β*-Catenin and CD34, and WB was used to detect the expression levels of *β*-Catenin and *p*-VEGFR2/VEGFR2 proteins. As shown in Figure 6(e), the WB results evidenced a significant increase in the protein levels of *β*-Catenin and *p*-VEGFR2/VEGFR2 in the *COMMD3*-overexpressed group compared to the controls, while they were suppressed in the *COMMD3*-RNA interference group. The outcomes of IHC staining also illustrated that the tumor staining intensity of *β*-Catenin and CD34 in the *COMMD3*-overexpressed group was higher than that of the control group, while the *COMMD3*-RNA interference group had lower intensity. Both *in vitro* and *in vivo* results verified cancer- and angiogenesis-promoting effects of *COMMD3* (Figure 7(b)).

### 3.13. COMMD3 Modulates Angiogenesis by Regulating HIF1*α*/VEGF/NF-*κ*B Signaling Pathways In Vivo

WB and IHC staining were used to determine the protein expression levels of HIF-1*α*, VEGF, and NF-*κ*B to further verify the mechanism of *COMMD3* regulation of angiogenesis of *COMMD3* in HCC subcutaneously transplanted nude mice. The results showed that for both WB (Figure 6(e)) and IHC staining (Figure 7(b)), *COMMD3* overexpression promoted the protein expression levels of HIF1*α*, VEGF, and NF-*κ*B *in vivo* compared to the controls, while *COMMD3*-RNA interference had the opposite effect. These findings indicated that *COMMD3* plays a positive regulatory role in the growth and angiogenesis of subcutaneous HCC xenografts, and its effects may be achieved by regulating the HIF1*α*/VEGF/NF-*κ*B signaling pathway.

## 4. Discussion

In this study, we successfully constructed two stable transgenic HCC cell lines (SK-Hep1 and Hep-3B) with *COMMD3* overexpression and *COMMD3* knockdown, respectively. We sequenced and compared the *COMMD3*-overexpressed SK-Hep1 cells, *COMMD3*-knockdown SK-Hep1 cells, and control SK-Hep1 cells, to show that changes in *COMMD3* expression were significantly related to angiogenesis. We also found that *COMMD3* overexpression induced the angiogenesis of HCC cells, while *COMMD3* knockdown inhibited HCC angiogenesis. Simultaneously, GSEA based on the sequencing results indicated that the NF-*κ*B and HIF1*α* pathways were most related to the differential expression of *COMMD3*. Therefore, our *in vitro* and *in vivo* analyses indicated the activating effect of *COMMD3* on the HIF1*α*/VEGF/NF-*κ*B pathway leading to the promotion of the angiogenesis and progression of HCC.

Recent studies have shown that the COMMD protein family is expected to become a potential therapeutic target for several cancers [13, 17, 18]. COMMD1 was the first member to be discovered and the most representative family member [19]. COMMD1 is a regulatory protein with several functions such as copper stabilization, iron transportation and secretion, oxidative stress response, DNA damage response, protein aggregation, NF-*κ*B- and hypoxia-mediated transcription, and carcinogenesis [12, 15, 20, 21]. Furthermore, COMMD3, COMMD4, and COMMD6 are suggested to be interacting with different NF-*κ*B subunits and participating in the inhibition of transcription processes mediated by NF-*κ*B. However, they all show relatively weaker effects compared to COMMD1 [22]. COMMD5, which is also known as HCaRG, is a hypertension-related calcium regulatory gene that inhibits the expansion of tumor cells [23–25]. COMMD7 is reported to have a suppressing effect on the expansion, migratory, and invasiveness capacities of hepatocarcinoma by regulating the chemokine CXCL10 [17, 26, 27]. Concurrently, it was found that COMMD7 regulates CXCL10 mainly through NF-*κ*B and reactive oxygen species (ROS) [28]. COMMD9 is also reported to have an inhibitory effect on the expansion and migration of non-small-cell lung cancer cells, blocking cells in the G1/S phase and inducing autophagy [18]. COMMD10 is a common member of the COMMD family which suppresses the invasiveness and metastatic properties of colorectal cancer cells through NF-*κ*B [29]. In contrast, COMMD2 and COMMD8 currently lack relevant reports on their functions, and limited research is available for COMMD3. Nevertheless, according to the literature, COMMD3 is expressed at a high level in prostate cancer, enhances migratory and invasiveness properties of tumor cells, and is also associated with tumor recurrence and low survival rate [30]. In recent years, research interest in the role of COMMD3 in HCC has steadily increased. By mining public information databases, Wang et al. [13] confirmed that COMMD3 is highly expressed in tissues of HCC patients and predicts poorer clinical outcomes. However, the experimental evidence on COMMD3 role in HCC, especially its mechanism, has remained uncertain.

**Figure 8:**
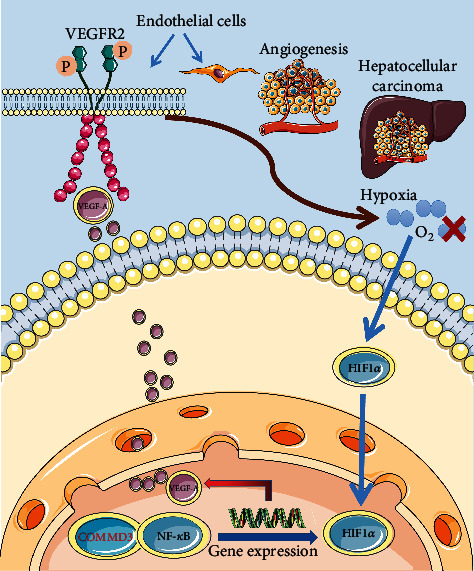
The schematic diagram of COMMD3 involved in HCC angiogenesis by regulating the HIF1*α*/VEGF/NF-*κ*B signaling pathway.

Previous studies have shown that NF-*κ*B seems to be inextricably linked with members of the COMMD family: it can positively or negatively regulate the NF-*κ*B signaling pathway in a variety of ways. NF-*κ*B is an essential factor in the process of inflammation and liver cell regeneration, and NF-*κ*B is usually overactivated in HCC. In patients with HCC and high NF-*κ*B activity, inflammation is difficult to control, pathological features are dangerous, and treatment effects are poor. Blocking the NF-*κ*B signaling pathway can inhibit tumor proliferation, indicating the importance of inhibiting NF-*κ*B activity in preventing the progression of HCC disease. In this study, we performed transcriptome sequencing and comparison of *COMMD3* overexpression and *COMMD3*-knockdown SK-Hep1 cell line with wild-type SK-Hep1 cell line. The findings indicate the involvement of *COMMD3* in the regulation of the NF-*κ*B signaling pathway. On the other hand, the survival and progression of solid tumors cause local hypoxia. Under hypoxic conditions, the NF-*κ*B pathway is activated and HIF-1*α* is accumulated. NF-*κ*B is a key transcription factor regulating HIF1*α*. This experimental study showed that *COMMD3* overexpression elevated the protein levels of *β*-Catenin, *p*-VEGFR2/VEGFR2, HIF1*α*, VEGF, and NF-*κ*B, whereas, after *COMMD3*-RNA interference, the expression levels of all these proteins were downregulated. Thus, we speculate that *COMMD3* interacts with NF-*κ*B in the local hypoxic microenvironment, where HIF1*α* enters the nucleus and accumulates and is activated by NF-*κ*B, *β*-Catenin expression is upregulated, VEGF secretion is induced, and VEGFR2 is phosphorylated. These processes ultimately promote angiogenesis in HCC, which assists with HCC invasion and metastasis, thereby causing more severe local tissue hypoxia and promoting tumor progression (Figure 8). Therefore, COMMD3 is expected to become a potential prognostic biomarker for HCC.

## 5. Conclusions

Our results demonstrate that the HIF1*α*/VEGF/NF-*κ*B signaling pathway participates in the effects of COMMD3 on HCC angiogenesis and progression both *in vitro* and *in vivo*. Further research in the future can be conducted to illustrate the potential of using COMMD3 as a therapeutic target in HCC.

## Data Availability

All data obtained or analyzed during this study are included within the article.

## Additional Points

*Reporting Checklist*. The authors have completed the ARRIVE reporting checklist.

## References

[1] Bray F., Ferlay J., Soerjomataram I., Siegel R. L., Torre L. A., Jemal A. (2018). Global cancer statistics 2018: GLOBOCAN estimates of incidence and mortality worldwide for 36 cancers in 185 countries. *CA: a Cancer Journal for Clinicians*.

[2] Llovet J. M., Castet F., Heikenwalder M. (2021). Immunotherapies for hepatocellular carcinoma. *Nature Reviews. Clinical Oncology*.

[3] Zhu A. X., Duda D. G., Sahani D. V., Jain R. K. (2011). HCC and angiogenesis: possible targets and future directions. *Nature Reviews. Clinical Oncology*.

[4] Burdette W. J. (1970). Carcinoma of the colon and antecedent epithelium. *Cancer Research*.

[5] Korbecki J., Simińska D., Gąssowska-Dobrowolska M. (2021). Chronic and cycling hypoxia: drivers of cancer chronic inflammation through HIF-1 and NF-*κ*B activation: a review of the molecular mechanisms. *International Journal of Molecular Sciences*.

[6] D'Ignazio L., Batie M., Rocha S. (2017). Hypoxia and inflammation in cancer, focus on HIF and NF-*κ*B. *Biomedicine*.

[7] Azoitei N., Becher A., Steinestel K. (2016). PKM2 promotes tumor angiogenesis by regulating HIF-1*α* through NF-*κ*B activation. *Molecular Cancer*.

[8] Qin S., Li A., Yi M., Yu S., Zhang M., Wu K. (2019). Recent advances on anti-angiogenesis receptor tyrosine kinase inhibitors in cancer therapy. *Journal of Hematology & Oncology*.

[9] Maine G. N., Burstein E. (2007). COMMD proteins: COMMing to the scene. *Cellular and Molecular Life Sciences*.

[10] Healy M. D., Hospenthal M. K., Hall R. J. (2018). Structural insights into the architecture and membrane interactions of the conserved COMMD proteins. *eLife*.

[11] Narindrasorasak S., Kulkarni P., Deschamps P., She Y. M., Sarkar B. (2007). Characterization and copper binding properties of human COMMD1 (MURR1). *Biochemistry*.

[12] Riera-Romo M. (2018). COMMD1: a multifunctional regulatory protein. *Journal of Cellular Biochemistry*.

[13] Wang X., He S., Zheng X. (2021). Transcriptional analysis of the expression, prognostic value and immune infiltration activities of the COMMD protein family in hepatocellular carcinoma. *BMC Cancer*.

[14] Rius J., Guma M., Schachtrup C. (2008). NF-*κ*B links innate immunity to the hypoxic response through transcriptional regulation of HIF-1*α*. *Nature*.

[15] van de Sluis B., Mao X., Zhai Y. (2010). COMMD1 disrupts HIF-1alpha/beta dimerization and inhibits human tumor cell invasion. *Journal of Clinical Investigation*.

[16] Zhu T., Cheng Z., Peng X., Xing D., Zhang M. (2021). HIF-1*α* RNAi combined with asparagus polysaccharide exerts an antiangiogenesis effect on hepatocellular carcinoma in vitro and in vivo. *Evidence-based Complementary and Alternative Medicine*.

[17] Zheng L., Deng C. L., Wang L. (2016). COMMD7 is correlated with a novel NF-*κ*B positive feedback loop in hepatocellular carcinoma. *Oncotarget*.

[18] Zhan W., Wang W., Han T. (2017). COMMD9 promotes TFDP1/E2F1 transcriptional activity via interaction with TFDP1 in non-small cell lung cancer. *Cellular Signalling*.

[19] Burstein E., Hoberg J. E., Wilkinson A. S. (2005). COMMD proteins, a novel family of structural and functional homologs of MURR1. *Journal of Biological Chemistry*.

[20] Zoubeidi A., Ettinger S., Beraldi E. (2010). Clusterin facilitates COMMD1 and I-kappaB degradation to enhance NF-kappaB activity in prostate cancer cells. *Molecular Cancer Research*.

[21] Taskinen M., Louhimo R., Koivula S. (2014). Deregulation of COMMD1 is associated with poor prognosis in diffuse large B-cell lymphoma. *PLoS One*.

[22] Liu Y. F., Swart M., Ke Y., Ly K., McDonald F. J. (2013). Functional interaction of COMMD3 and COMMD9 with the epithelial sodium channel. *American Journal of Physiology. Renal Physiology*.

[23] Solban N., Jia H. P., Richard S. (2000). HCaRG , a novel calcium-regulated gene coding for a nuclear protein, is potentially involved in the regulation of cell proliferation. *The Journal of Biological Chemistry*.

[24] Chen B. L., Yu J., Zeng Z. R. (2008). Rosiglitazone suppresses gastric carcinogenesis by up-regulating HCaRG expression. *Oncology Reports*.

[25] Matsuda H., Campion C. G., Fujiwara K. (2017). HCaRG/COMMD5 inhibits ErbB receptor-driven renal cell carcinoma. *Oncotarget*.

[26] Zheng L., Liang P., Li J. (2012). ShRNA-targeted COMMD7 suppresses hepatocellular carcinoma growth. *PLoS One*.

[27] You N., Li J., Huang X. (2017). COMMD7 promotes hepatocellular carcinoma through regulating CXCL10. *Biomedicine & Pharmacotherapy*.

[28] You N., Li J., Huang X. (2018). COMMD7 activates CXCL10 production by regulating NF-*κ*B and the production of reactive oxygen species. *Molecular Medicine Reports*.

[29] Yang S. S., Li X. M., Yang M. (2017). FMNL2 destabilises COMMD10 to activate NF- *κ* B pathway in invasion and metastasis of colorectal cancer. *British Journal of Cancer*.

[30] Umbreen S., Banday M. M., Jamroze A. (2019). COMMD3: BMI1 fusion and COMMD3 protein regulate C-MYC transcription: novel therapeutic target for metastatic prostate cancer. *Molecular Cancer Therapeutics*.

